# Inverse Phosphatidylcholine/Phosphatidylinositol Levels as Peripheral Biomarkers and Phosphatidylcholine/Lysophosphatidylethanolamine-Phosphatidylserine as Hippocampal Indicator of Postischemic Cognitive Impairment in Rats

**DOI:** 10.3389/fnins.2018.00989

**Published:** 2018-12-21

**Authors:** Angelica Maria Sabogal-Guáqueta, Javier Gustavo Villamil-Ortiz, Julian David Arias-Londoño, Gloria Patricia Cardona-Gómez

**Affiliations:** ^1^Neuroscience Group of Antioquia, Cellular and Molecular Neurobiology Area – School of Medicine, Sede de Investigación Universitaria (SIU), University of Antioquia, Medellin, Colombia; ^2^Department of Systems Engineering, University of Antioquia, Medellín, Colombia

**Keywords:** global ischemia, cognitive impairment, phospholipid profile, biomarkers, serum, hippocampus

## Abstract

Vascular dementia is a transversal phenomenon in different kinds of neurodegenerative diseases involving acute and chronic brain alterations. Specifically, the role of phospholipids in the pathogenesis of dementia remains unknown. In the present study, we explored phospholipid profiles a month postischemia in cognitively impaired rats. The two-vessel occlusion (2-VO) model was used to generate brain parenchyma ischemia in adult male rats confirmed by alterations in myelin, endothelium, astrocytes and inflammation mediator. A lipidomic analysis was performed via mass spectrometry in the hippocampus and serum a month postischemia. We found decreases in phospholipids (PLs) associated with neurotransmission, such as phosphatidylcholine (PC 32:0, PC 34:2, PC 36:3, PC 36:4, and PC 42:1), and increases in PLs implied in membrane structure and signaling, such as lysophosphatidylethanolamine (LPE 18:1, 20:3, and 22:6) and phosphatidylserine (PS 38:4, 36:2, and 40:4), in the hippocampus. Complementarily, PC (PC 34:2, PC 34:3, PC 38:5, and PC 36:5) and ether-PC (ePC 34:1, 34:2, 36:2, 38:2, and 38:3) decreased, while Lyso-PC (LPC 18:0, 18:1, 20:4, 20:5, and LPC 22:6) and phosphatidylinositol (PI 36:2, 38:4, 38:5, and 40:5), as neurovascular state sensors, increased in the serum. Taken together, these data suggest inverse PC/LPC-PI levels as peripheral biomarkers and inverse PC/LPE-PS as a central indicator of postischemic cognitive impairment in rats.

## Introduction

Cognitive impairment and dementia are common phenomena induced by acute and chronic brain injury, including Alzheimer’s disease, stroke, and traumatic brain injury (TBI) (Nucera and Hachinski, 2018). Additionally, these conditions are predisposed to or worsened by poor lifestyles, including obesity, metabolic disorders, sedentary habits, smoking, and cardiocerebrovascular diseases, among others (Moskowitz et al., 2010). Long-term deprivation of oxygen and glucose via hypoperfusion or vasoconstriction of small vessels generates neurovascular unit injury, which is associated with vascular dementia after focal or global cerebral ischemic injury in response to cardiac arrest, coronary artery bypass surgery, cardiorespiratory failure, and other conditions due to drastic reductions in blood flow to the brain (Llinas et al., 2000; Vijayan et al., 2017).

In particular, the incidence of cerebrovascular disease, the third leading cause of death and the first leading cause of physical and mental disability worldwide, is increasing in developing countries (Cardona-Gómez and Lopera, 2016). Particularly, brain ischemia is caused by the occlusion of blood vessels, depriving of oxygen and glucose, resulting in an energy failure that alters mitochondrial ATP synthesis and upregulates the production of oxidative stress, free radicals and lipid peroxidation. Also,, the activation of dopamine and glutamate, as excitatory neurotransmitters, induces intracellular calcium overload, metabolic dysfunction and acidosis (Moskowitz et al., 2010). The increase in intracellular calcium causes activation of enzymes involved in lipid metabolism, such as “sphingomyelinases and phospholipases A2, C, and D, that, in turn, promote the release of second messengers, such as diacylglycerol (DAG), phosphatidic acid (PA), and arachidonic acid (AA), involved in inflammation, excitotoxicity and other cell death pathways” (Phillis and O’Regan, 2004; Tian et al., 2009). However, biomarkers supporting the clinical diagnosis of stroke are in development, and the development of biomarkers used to diagnosis the risk of dementia after stroke and its prevention is a true challenge.

In regard to lipids, these compounds are diverse, complex, and their functions depend on cellular distribution. Lipids are crucial in the homeostasis of cell membrane structure and act as signaling molecules and modulators in the central nervous system (CNS) (Martinez-Gardeazabal et al., 2017). Interestingly membrane lipids can be damaged by lipolysis under ischemia and by peroxidation of polyunsaturated fatty acids (PUFAs) during reperfusion (Schaller and Graf, 2004). Particularly, phospholipids, which are known for their high concentrations in the brain, play an important role both in normal neuronal activity and in pathological processes, even in those associated with memory impairment (Miyawaki et al., 2016). Therefore, we focused on elucidating the changes in lipid profiles of the hippocampus and serum of rats with cognitive impairment induced by global ischemia, as a continue of our previous studies (Marosi et al., 2006).

## Materials and Methods

### Animal Procedures

“All of the animal procedures were performed in accordance with the ARRIVE guidelines, the Guide for the Care and Use of Laboratory Animals, 8th edition published by the National Institutes of Health (NIH) and the Colombian standards (law 84/1989 and resolution 8430/1993). These procedures were approved by the Ethics Committee for Animal Experimentation of the University of Antioquia, Medellin, Colombia.

Male Wistar albino rats from our in-house, pathogen-free colony in the vivarium at SIU (Sede de Investigación Universitaria), University of Antioquia, Medellin, Colombia were kept on a 12:12 h dark/light cycle and received food and water *ad libitum*. Special care was taken to minimize animal suffering and to reduce the number of animals used. Three-month-old rats weighing 400–450 g were used. The rats were randomly divided into two groups, namely, the control and ischemic groups. Nine (9) rats were used per experimental group for behavioral, lipidomic and immunostaining evaluation (Marosi et al., 2006).

### Global Cerebral Ischemia (2 VO)

“The animals were anesthetized using ketamine (60 mg/kg) and xylazine (5 mg/kg) and received a 2–4% isofiurane and 96% oxygen mixture via an inhalation anesthesia machine. A variation of the global cerebral ischemic model was implemented, involving a 2-vessel occlusion (2-VO; (Marosi et al., 2006). The right common carotid artery (CCA) was permanently occluded using a 6.0-gauge nylon suture (Corpaul, Bogota, Colombia), and the left CCA was obstructed for 20 min using a vascular clip. After the 20 min, the vascular clip was removed to allow reperfusion. Sham control rats underwent the same procedure without the CCA occlusion. The animals were sacrificed a month postischemia for lipid analyses” (Becerra-calixto and Cardona-gómez, 2017).

### Immunochemistry and Immunofluorescence

Twenty four hours after the last behavioral test, animals were perfused intracardially with paraformaldehyde at 4%. Brains were removed and postfixed 48 h. Coronal sections (50 mm) obtained from vibratome were permeabilized, with 0.3% Triton X-100 and blocked with 1% BSA in PBS, using a previously described protocols for immunohistochemistry and immunofluorescence (Marosi et al., 2006), for the following evaluated primary antibodies: anti-NeuN (mouse monoclonal, Millipore, 1:500) anti-GFAP (monoclonal anti-glial fibrillary acidic protein, Sigma, 1:1000), anti-PECAM-1 (rabbit Platelet Endothelial Cell Adhesion Molecule 1, Abcam, 1:500), anti-myelin PLP (rabbit, myelin Proteolipid protein, Abcam, 1:200), COX-2 (rabbit, Cyclooxygenase 2, Abcam, 1:500). For immunofluorescence tissue, we incubated for 90 min at room temperature with mouse Alexa Fluor 488- or Alexa Fluor 594- conjugated anti-rabbit secondary antibodies (1:1000; Molecular Probes, Eugene, OR, United States). The tissues incubated in the absence of primary antibody did not display immunoreactivity.

### Morris Water Maze Test

“Nineteen days after ischemia, the animals were evaluated in the Morris water maze (MWM) teste during 10 days (*n* = 9 per group). The test was performed using a previously described method (Becerra-calixto and Cardona-gómez, 2017). Briefly, a black plastic tank was filled with water (22 ± 2°C), and visual cues around the room remained in a fixed position throughout the experiment. The hidden platform (12 cm in diameter) was submerged 3 cm below the water level during spatial learning and 1.5 cm above the surface of the water during the visible session. Six sessions or trials were performed. Each session consisted of four successive subtrials (30 s intertrial interval), and each subtrial began with the rat being placed pseudorandomly in one of four starting locations. Then, the animals were provided with a 48 h retention period, followed by a probe trial of spatial reference memory, in which the animals were placed in the tank without the platform for 90 s. The latency to reach the exact former location of the platform was recorded during the probe trial. Later, the platform was moved to a new location and the ability of the animals to learn the new location was measured by determining the latency in 4 sessions conducted in the same manner as the learning phase. The latency to reach the platform was evaluated using a visible platform to control for any differences in visual-motor abilities or motivation between the experimental groups; the animals that could not perform the task were excluded. An automated system (Viewpoint, Lyon, France) recorded the behavior of the animals” (Marosi et al., 2006).

### Tissue Preparation and Lipid Extraction

A month postischemia, four (4) “animals were sacrificed via decapitation, and the hippocampus of each rat was dissected, immediately frozen in liquid nitrogen and stored at 80°C until analysis. We performed the same procedure to obtain serum samples. The total lipids from the hippocampus and serum were extracted according to the FOLCH technique (Jordi Folch, 1957) using a mixture of 2 mL of chloroform (CHCl3) and 1 mL of methanol (MeOH) in a 2:1 (v/v) ratio. Then, 0.005% butylated hydroxytoluene (BHT) was added, and this mixture was used to homogenize the hippocampus. Subsequently, 1 mL of 0.9% NaCl was added, and the mixture was centrifuged at 3000 rpm for 3 min. The organic layer (lower layer) was removed and transferred to a new glass tube. The solvents were evaporated, and the extract was lyophilized to remove excess humidity. Finally, the lipid composition was analyzed via mass spectrometry” (Villamil-Ortiz et al., 2016).

### Mass Spectrometry

“An automated ESI-MS/MS approach was used, and data acquisition and analysis were carried out at the Kansas Lipidomics Research Center using an API 4000 TM and Q-TRAP (4000Qtrap) detection system as described previously (Villamil-Ortiz et al., 2016). This protocol allowed the detection and quantification of low concentrations of the polar lipid compounds. The molecules were determined by the mass/charge ratios, which were compared with the respective internal standard to determine which species of lipids were present in the evaluated extract: 0.30 nmol 14:0 lysoPG, 0.30 nmol 18:0 lysoPG, 0.30 nmol di 14:0 PG, 0.30 nmol 14:0-lysoPE, 0.30 nmol 18:0-lysoPE, 0.60 nmol 13:0-lysoPC, 0.60 nmol 19:0-lysoPC, 0.60 nmol di 12:0-PC, 0.60 nmol di 24:1-PC, 0.30 nmol 14:0 lysoPA, 0.30 nmol 18:0 lysoPA, 0.30 nmol di14:0-PA, 0.30 nmol di20:0 (phytanoyl)-PA, 0.20 nmol di 14:0-PS, 0.20 nmol di Phy PS, 0.28 nmol 16:0-18:0 PI, and 0.10 nmol di 18:0-PI. The system detected a total of 12 different lipid species and their respective subspecies, which were identified by the number of carbons and the degree of unsaturation in the chain. The lipid concentration was normalized according to the molar concentration across all species for each sample, and the final data are presented as the mean mol%” (Villamil-Ortiz et al., 2016).

### Profile of Other Lipid Fractions

Lipids were extracted from the hippocampus and serum using the Folch method. The solid-phase extraction (SPE) as described Bermudez-Cardona et al (Bermúdez-cardona and Velásquez-rodríguez, 2016), was used to separate cholesterol esters (CE), triglycerides (TG), and free fatty acids (FFA).

### Statistical Analysis

The behavioral test comparisons between two groups were performed using Student’s *t*-tests for parametric data or Mann–Whitney tests for nonparametric data. “The lipid levels of each sample were calculated by summing the total number of moles of all lipid species measured and then normalizing that total to mol%. Comparisons between groups were assessed either by one-way ANOVA, followed by the Tukey *post hoc* test, or the Kruskal–Wallis test, depending on the homoscedasticity and normality of the experimental data. Multivariate statistics were performed using principal component analysis (PCA) and a partial least squares discriminant analysis (PLS-DA)” (Barker and Rayens, 2003). “The PLS-DA was included because this analysis is particularly suitable for the analysis of datasets with a small number of samples and a large number of variables. The PLS-DA was carried out using the protocols described previously by our laboratory” (Villamil-Ortiz et al., 2016). The data are expressed as the mean the standard error of the mean. The statistical significance is indicated in the figures and tables.

## Results

### Morphological Changes, Cognitive Impairment, and Hippocampal Phospholipid Profile Changes a Month Postischemia

**FIGURE 1:**
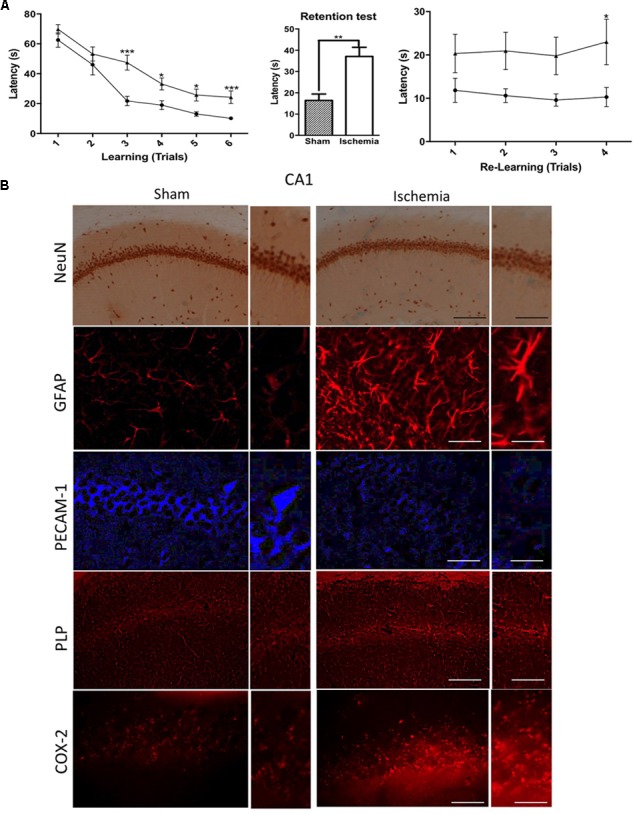
Morphological alterations and cognitive impairment in the rat hippocampus after transient global ischemia. **(A)** The learning and memory task performance was evaluated with the Morris water maze on day 19, starting with the learning test and the first position of the platform. The retention test was conducted after 48 h without the platform. The transference test included the second position of the platform. Data are expressed as group means ± SEM. ^∗^*p* < 0.05, ^∗∗^*p* < 0.01, and ^∗∗∗^*p* < 0.001; *n* = 9 animals/group. **(B)** Immunostaining in pyramidal cell layer in the hippocampus with Neuronal nuclei (NeuN) immunohistochemistry; glial fibrillary acidic protein (GFAP), Platelet Endothelial Cell Adhesion Molecule 1 (PECAM-1), myelin Proteolipid protein (PLP), Cyclooxygenase 2 (COX-2) immunofluorescences. *n* = 5. NeuN and PLP, Magnification: 10×, scale bar: 100 μm; Insert: Magnification 40×; scale bar = 50 μm; GFAP, PECAM, and COX-2. Magnification 20×, scale bar: scale bar = 50 μm; Insert: 60×, scale bar: 15 μm.

The abilities of spatial learning and memory were examined by using the Morris water maze test. We found that, even a month postischemia, ischemic rats significantly increased their latencies to locate the platform in the MWM from trial 3 to trial 6 (Figure 1A). In the memory test, it was found that rats with global ischemia showed significant deficits in locating the submerged escape platform compared with sham rats (Figure 1A). In addition, during the relearning test, ischemic rats exhibited cognitive impairment a month postischemia, and the escape latencies were significantly lower compared with those of the sham group (Figure 1A). Which was supported by the morphological alterations of the hippocampus, although there was not a clear neuronal loss, we detected hypertrophic astrocytes with thickened processes and enlarged cell bodies (Figure 1B). PECAM-1, a cell adhesion marker, suggested disruption of the parenchima, PLP, a protein involved in the production of myelin, presented aggregation at the CA1, in addition to the increased presence of COX-2, inflammatory marker, suggesting the structural damage in the hippocampus at 1 month post global ischemia respect to healthy rats (Figure 1B).

**FIGURE 2:**
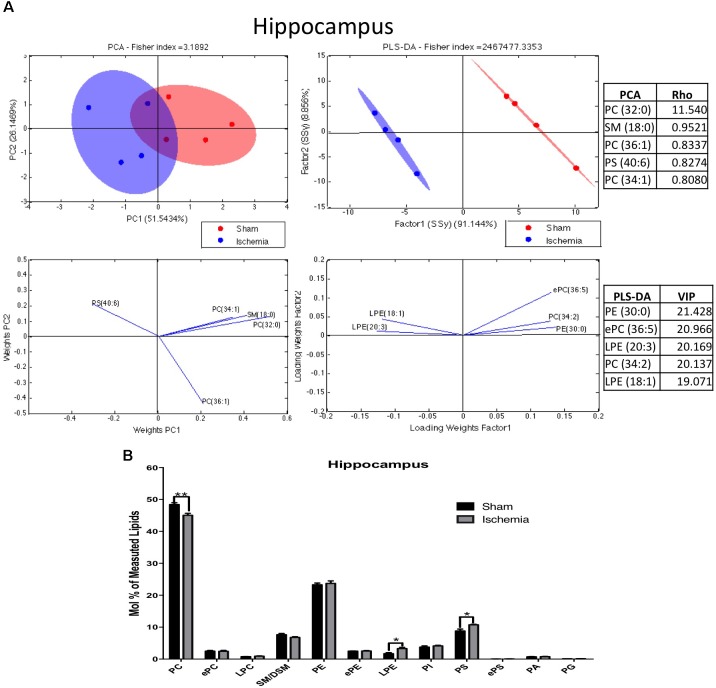
Phospholipid composition in the hippocampus after 1 month postischemia. **(A)** Multivariate analyses of the lipid profiles in the hippocampus. PCA and PLS-DA were performed to discriminate between the lipid classes. The left panel illustrates the factor loadings for PC1 and PC2 with the indices of variance explained for each component. The right panels show the factor score plots for the PLS-DA. **(B)** The lipid class profiles are expressed as % mol composition. All lipid species are represented as the means ± SEM. Data for ischemic group were significantly different from those of the control group (^∗^*p* < 0.05, ^∗∗^*p* < 0.01; Student’s *t*-test for parametric data or the Mann–Whitney test for nonparametric data, *n* = 4 per group).

On the other hand, 311 species of phospholipids were evaluated via mass spectrometry in the hippocampus a month postglobal ischemia. At a glance, the analyses indicate that the changes seem to be related to the pathological condition. The lipid profiles of both groups show that they were primarily composed of high-abundance glycerophospholipids, such as PC (48.4 and 47%), PE (23.2 and 23.4%), PS (8.8 and 9.7%), and PI (3.8 and 3.9%); sphingolipids, such as SM-DSM (7.5 and 7.2%); low-abundance ether phospholipids, such as ePC (2.5 and 2.48%), ePE (2.48 and 2.5%), and ePS (0.02 and 0.03%); lysophospholipids, such as LPE (1.68 and 2.35%) and LPC (0.77 and 0.83%); and glycerophospholipids, such as PA (0.74 and 0.76%) and PG (0.11 and 0.12%) (Figures 2A,B).

The results of the PCA of the detected lipids indicated that nearly 77% of the total variance might be explained by the first two principal components (PC1 and PC2) (Figure 2A). The most relevant variables for these two components were related to reduced PC and increased PS subclasses (Figure 2B). The main Rho indices were PC 32:0, 34:1, and 36:1. PC 34:1 was composed of saturated palmitic acid (16:0) and oleic acid (18:1), while PC 32:0 was composed of two palmitic acids. In addition, PS 40:6, conformed by stearic acid (18:0) and docosahexaenoic acid (DHA) (22:6), was represented in the PCA. Complementarily, the PLS-DA showed ellipsoids that were completely different in terms of location; this could explain the reason that the control groups had a more diverse lipid composition compared with the ischemic group. The main changes were shown in LPE 18:1, 20:3, PE 30:0, PC 34:2, and ePC 36:5. Together these findings, based on the abundance by PCA and separability by PLS-DA, suggest decreased PC and increased PS and LPE composed of imbalanced saturated fatty acid (SFA), monounsaturated FA (MFA) and in polyunsaturated FA (PUFA) after ischemia.

### Inverse PC and LPE/PS Levels in Postischemic Hippocampus of Rats

PCs are a class of 1,2-diacylglycerophospholipids that are essential components of cell membranes and have structural roles defined primarily by chain length (Billah and Anthes, 1990; Whiley et al., 2014). In previous studies using tMCAO (transient middle cerebral artery occlusion), it has been described that several PC species and sphingomyelin (SM) were significantly decreased after infarction in the cerebral cortex; in the same manner, LPCs were elevated in the tissue (Wang et al., 2010).

**FIGURE 3:**
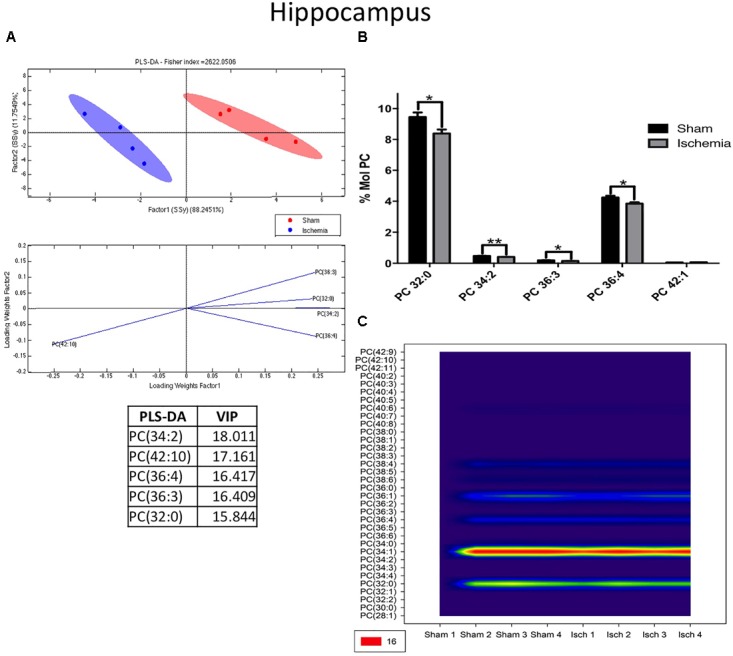
Changes in phosphatidylcholine in the hippocampus after global ischemia. **(A)** Multivariate analyses: PLS-DA used to discriminate between the lipid classes in the hippocampus showing the factor score plots. **(B)** The PC profile is expressed as % mol composition. **(C)** Contour plots of the more influential subclasses of PC (variables) in the discriminant analysis for each evaluated variable. Data from the ischemic group were significantly different from those of the control group and are represented as the means ± SEM (^∗^*p* < 0.05, ^∗∗^*p* < 0.01, and ^∗∗∗^*p* < 0.001; Student’s *t*-test for parametric data or the Mann–Whitney test for nonparametric data, *n* = 4 per group).

We detected that global ischemia had a regulatory effect on the lipid profiles of PC and LPE from the hippocampus. The PLS-DA showed that the ischemic group had ellipsoids in different spaces, explained by the first component in 88% and the second one in 11% approximately (Figure 3A). In general, the PC levels decreased (^∗^*p* < 0.05) in the ischemic groups compared with their counterpart in the sham control group (Figure 2B). Four PC subspecies showed significant reductions in global ischemia compared with those in the controls: 32:0 (16:0/16:0 - *p* < 0.05), 34:2 (16:1/18:1 (*p* < 0.01), 36:3 (18:1/18:2 - *p* < 0.05), and PC 36:4 (18:2/18:2) (Figures 3B,C). In general, considering that PC as the more abundant PL detected in the profile, those results were supported by the tendency and significant reduction of C.16:0 and C 18:0 at the total Free fatty acid (FFA) detected at the hippocampus and inversely C16:0 and C 18:0 were increased in the content of total triglycerides by the global ischemia. However, the CE did not change, lignoceric acid (24:0) FFA increased and a generalized reduction of oleic acid (18:1) was observed in the three analyzed fractions in the ischemic hippocampus (Supplementary Figures S1A–C).

**FIGURE 4:**
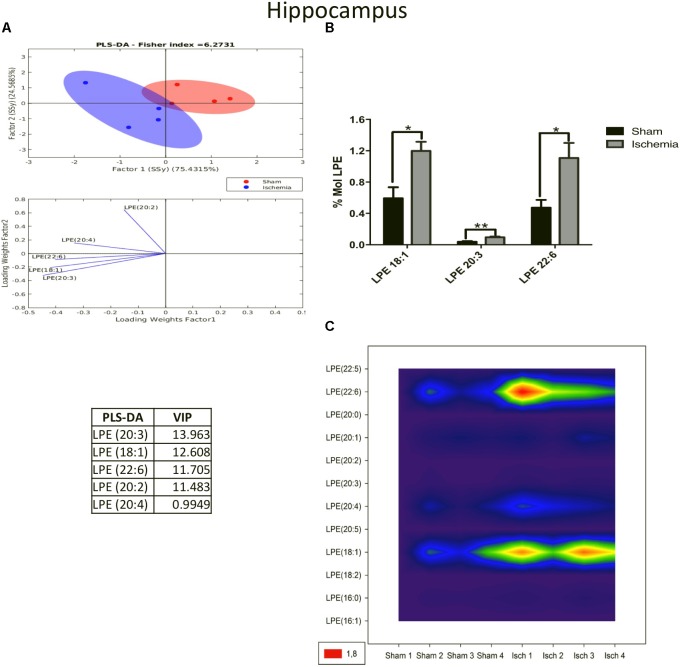
Increase in LPE species in the hippocampus after global cerebral ischemia. **(A)** Multivariate analyses: PLS-DA used to discriminate between the lipid classes in the hippocampus showing the factor score plots. **(B)** The LPE profile is expressed as % mol composition. **(C)** Contour plots of the more influential subclasses of LPE (variables) in the discriminant analysis for each evaluated variable. Data from the ischemic group were significantly different than those from the control group and are represented as the means ± SEM (^∗^*p* < 0.05, ^∗∗^*p* < 0.01, and ^∗∗∗^*p* < 0.001; Student’s *t*-test for parametric data or the Mann–Whitney test for nonparametric data, *n* = 4 per group).

On the other hand, LPE, a plasmalogen derived from phosphatidylethanolamine (PE), had significant higher levels (*p* < 0.05) in the ischemia group than in the sham group. The PLS-DA demonstrated that the ischemic group occupied a close area with respect to that of the sham group, having a small overlapping area, however, the separability was explained by the first component at a rate of 75.43% (Figure 4A). When the most differential and relevant phospholipidic species was LPE 18:1, 20:3, and 22:6 increased in the ischemic group (Figures 4A,B). These results were supported by the contour graphic, which showed increasing levels in these PL subclasses in the global ischemia group, mainly LPE 22:6 and 18:1 (Figure 4C).

**FIGURE 5:**
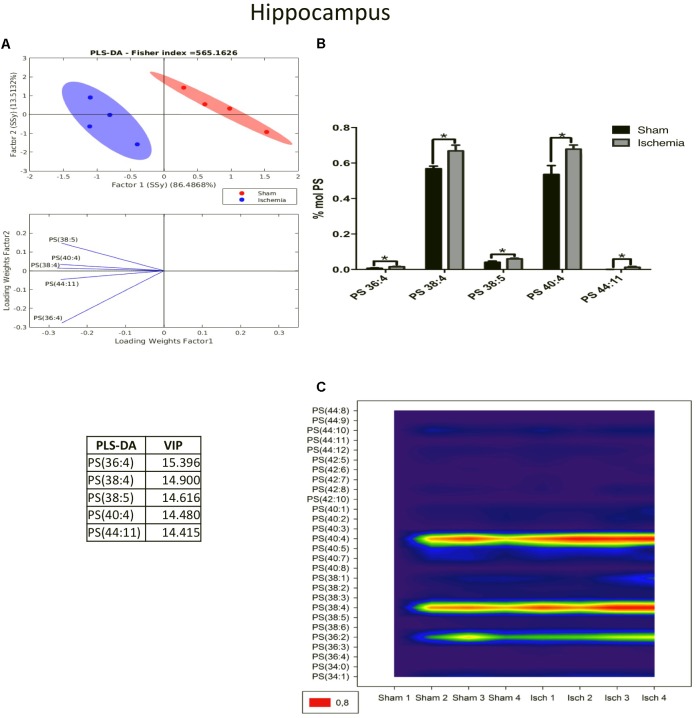
PS species increased in the ischemic hippocampus. **(A)** Multivariate analyses: PLS-DA used to discriminate between the lipid classes in the hippocampus showing the factor score plots. **(B)** The PS profile is expressed as % mol composition. **(C)** Contour plots of the more influential subclasses of PS (variables) in the discriminant analysis for each evaluated variable. Data from the ischemic group were significantly different from those from the control group and are represented as the means ± SEM (^∗^*p* < 0.05 Student’s *t*-test for parametric data or the Mann–Whitney test for nonparametric data, *n* = 4 per group).

Complementarily, PS modulates the binding properties of glutamate receptors involved in neurotransmission and long-term potentiation in the brain (Farooqui and Horrocks, 2007). The PLS-DA showed a different distribution of the ischemic group relative to that of the sham group in the PCA graph. These data showed that the main changes occurred in the subspecies of PC 36:4, 38:4, 40:4, 38:5, and 44:11, as was indicated by the VIP index (Figure 5A). Most of these subspecies are composed of arachidonic acid (20:4), a proinflammatory molecule. Given that their levels significantly increased in the ischemic group (^∗^*p* < 0.05) (Figure 5B), this finding was in agreement with the high abundance shown in the counter plot graphic mainly by PS 40:4, 38:4, and 36:2 (Figure 5C).

### Phospholipidic Profile Changes in the Serum a Month Postischemia in Rats

**FIGURE 6:**
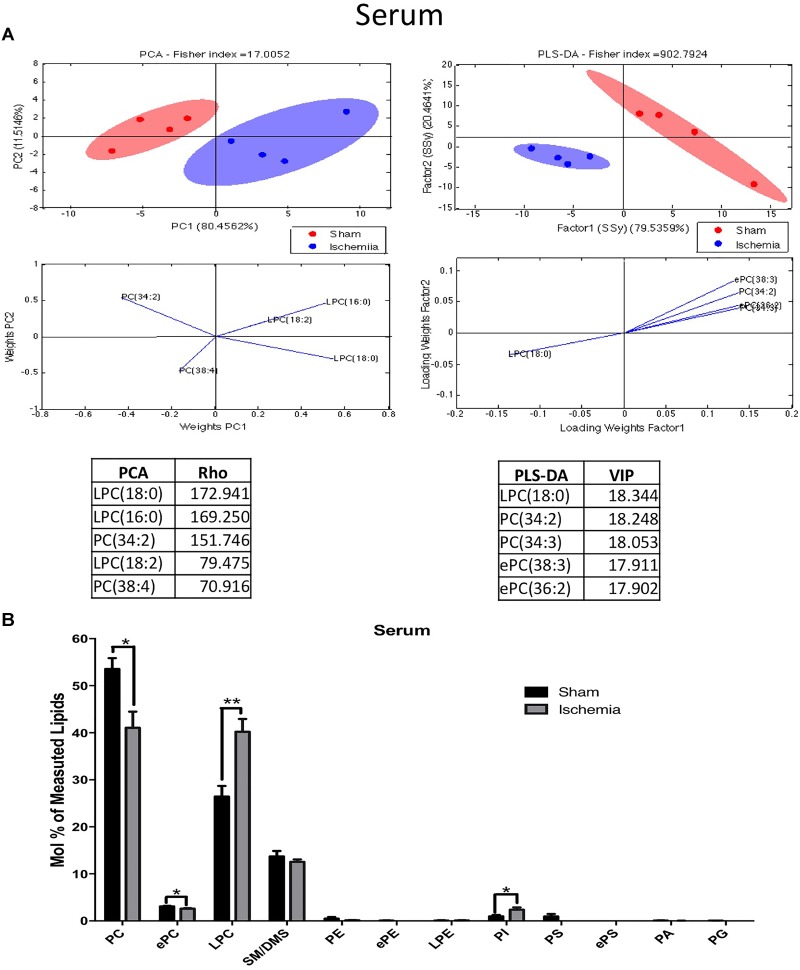
Altered phospholipid composition in the serum 1 month postischemia. **(A)** Multivariate analyses of the lipid profiles in the serum. PCA and PLS-DA were used to discriminate between the lipid classes. The left panel illustrates the factor loadings for PC1 and PC2 with the indices of variance explained for each component. The right panels show the factor score plots for the PLS-DA. **(B)** The lipid class profiles are expressed as % mol composition. All lipid species are represented as the means ± SEM. Data from the ischemic group were significantly different from those from the control group (^∗^*p* < 0.05, ^∗∗^*p* < 0.01, Student’s *t*-test for parametric data or the Mann–Whitney test for nonparametric data, *n* = 4 per group).

Evaluation of the lipid profiles of serum in ischemic rats with cognitive impairment a month postischemia could suggest potential biomarkers. The results of the PCA of the detected lipids indicated that nearly 92% of the total variance might be explained by the first two principal components (PC1 and PC2). The distribution pattern in the plane showed divergent distributions between the ischemic and sham groups in the lipid profiles of the serum (Figure 6A). Complementarily, the PLS-DA confirmed a displacement in the left quadrant of the ischemic group, while the sham group occupied a different region on the right side. PCA showed abundant and differential locations of LPC and PC between the control and ischemic groups, with the main divergent phospholipid species being LPC 18:0, 16:0 and PC 34:2 with a Rho of 172.9, 169.25, and 151.74, respectively. While PLS-DA also identified LPC 18:0 and PC 34:2 as the more discriminant species, with a VIP of 18.34 and 18.24, respectively, between others explained by the first component in approximately 80% (Figure 6A).

The lipid profiles of both groups showed that they were primarily composed of high-abundance glycerophospholipids, such as PC (53.3 and 47.9%) and LPC (26.43 and 23.55%); sphingolipids, such as SM-DSM (13.7 and 13.2%); low-abundance ether phospholipids, such as ePC (3.07 and 2.85%), ePE (0.05 and 0.03%), and ePS (0.008 and 0.004%); PE (0.48 and 0.32%); PS (0.92 and 0.51%); PI (0.94 and 1.58%); LPE (0.068 and 0.074%); PA (0.05 and 0.04%) and PG (0.03 and 0.02%) (Figure 6B). In addition, these analyses showed that the main changes in serum were due to inverse levels of phosphatidylcholine and its plasmalogen, lysophosphatidylcholine, and the intermediate ePC species; together, a significant increase in PI subspecies occurred in the serum of the ischemic group.

### Inverse PC and LPC-PI Serum Levels in Postischemic and Cognitively Impaired Rats

**FIGURE 7:**
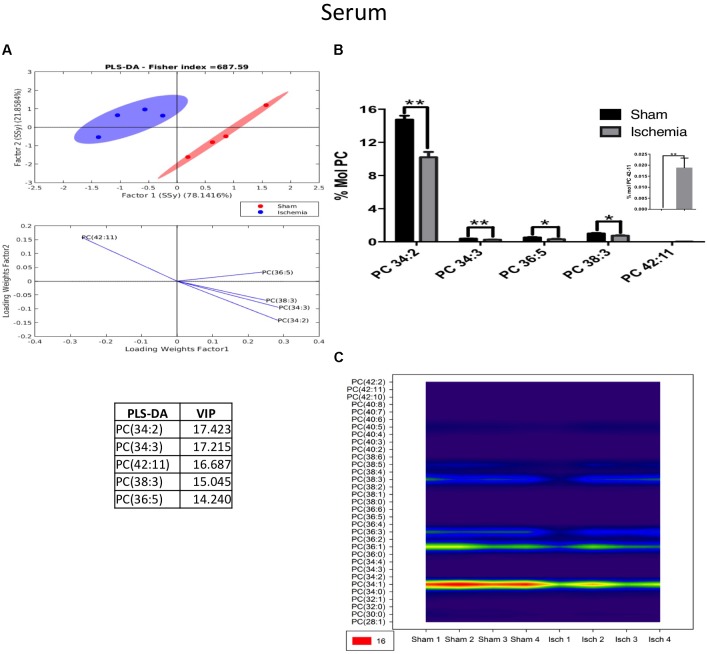
The majority of the species of PC decreased in the serum of postischemic rats. **(A)** Multivariate analyses: PLS-DA analysis used to discriminate between the lipid classes in the serum showing the factor score plots. **(B)** The PC profile is expressed as % mol composition. **(C)** Contour plots of the more influential subclasses of PC (variables) in the discriminant analysis for each evaluated variable. Data from the ischemic group were significantly different from those from the control groups and are represented as the means ± SEM (^∗^*p* < 0.05, ^∗∗^*p* < 0.01, and ^∗∗∗^*p* < 0.001; Student’s *t*-test for parametric data or the Mann–Whitney test for nonparametric data, *n* = 4 per group).

**FIGURE 8:**
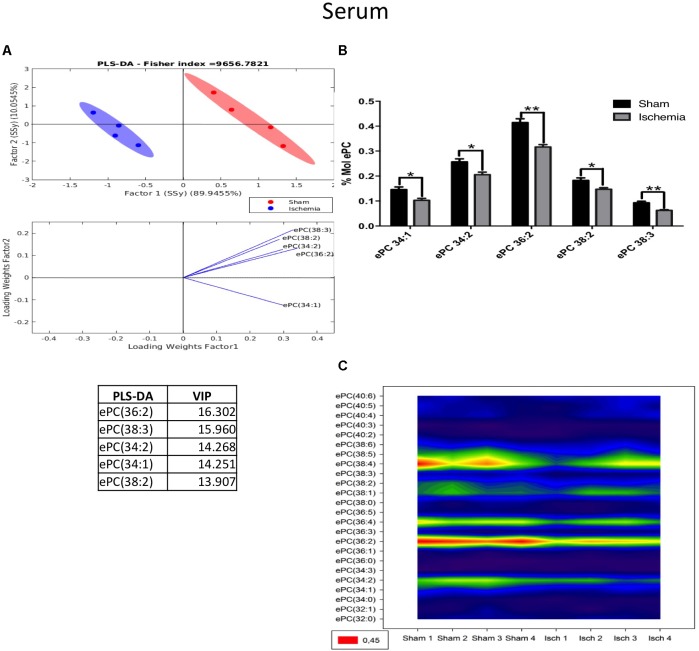
Changes in ePC in the serum of postischemic rats. **(A)** Multivariate analyses: PLS-DA analysis used to discriminate between the lipid classes in the serum showing the factor score plots. **(B)** The ePC profile is expressed as % mol composition. **(C)** Contour plots of the more influential subclasses of ePC (variables) in the discriminant analysis for each evaluated variable. Data from the ischemic group were significantly different from those from the control group and are represented as the means ± SEM (^∗^*p* < 0.05, ^∗∗^*p* < 0.01, and ^∗∗∗^*p* < 0.001; Student’s *t*-test for parametric data or the Mann–Whitney test for nonparametric data, *n* = 4 per group).

Our results indicated that lipid phosphatidylcholine molecule subspecies, such as PC 34:2, PC 34:3, PC 36:5, and PC 38:3, significantly decreased in the serum of the ischemic group, showing a notable separability (Figures 7A,B) and reduced abundance (Figure 7C) relative to those in the sham group (Figure 7). Similarly, we observed a reduction in ePC, as shown in Figure 8A, and the PLS-DA showed that the sham and ischemic rats had different patterns of distribution, and these changes were reflected by the following subspecies: ePC 36:2, ePC 38:3, ePC 34:2, ePC 34:1, and ePC 38:2 (Figure 8A), all of which were significantly reduced with respect to the levels observed in the control group (Figure 8B) and supported by the counter graph (Figure 8C). Those results could be supported by a general reduction of C 18 in the total FFA in serum (Supplementary Figures S1D–F), and no changes were detected in CE.

**FIGURE 9:**
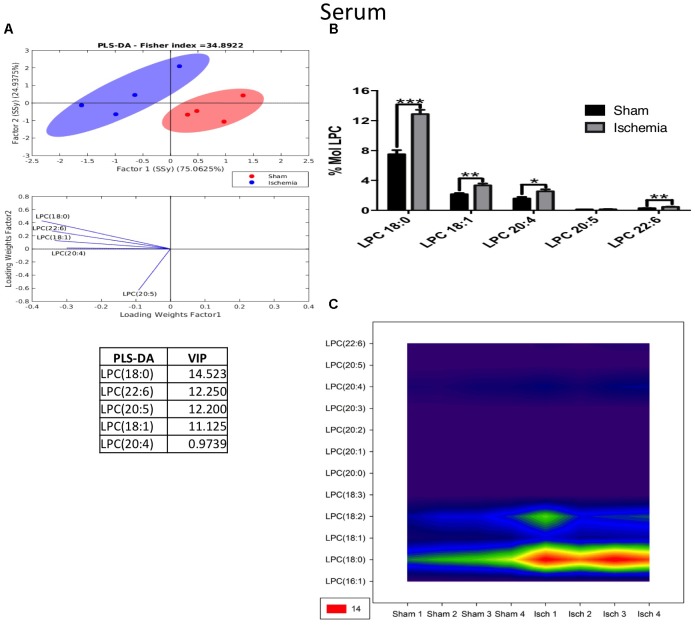
Altered LPC in the serum after global ischemia. **(A)** Multivariate analyses: PLS-DA analysis used to discriminate between the lipid classes in the serum showing the factor score plots. **(B)** The LPC profile is expressed as % mol composition. **(C)** Contour plots of the more influential subclasses of LPC (variables) in the discriminant analysis for each evaluated variable. Data from the ischemic group were significantly different from those from the control group and are represented as the means ± SEM (^∗^*p* < 0.05, ^∗∗^*p* < 0.01, and ^∗∗∗^*p* < 0.001; Student’s *t*-test for parametric data or the Mann–Whitney test for nonparametric data, *n* = 4 per group).

Inversely, LPC was shown to have increased in ischemic rats 1 month postischemia. The PLS-DA showed that ischemic and sham ellipsoids occupied different locations, supporting their different profiles (Figure 9A). The main subspecies that increased were LPC 18:0, LPC 22:6, LPC 20:5, LPC 18:1, and LPC 20:4 (Figures 9A,B) in cases when LPC 18:0, which is a fatty acid involved in inflammatory processes in cerebrovascular disease, detected at a high level also by the counter graph (Figure 9C).

**FIGURE 10:**
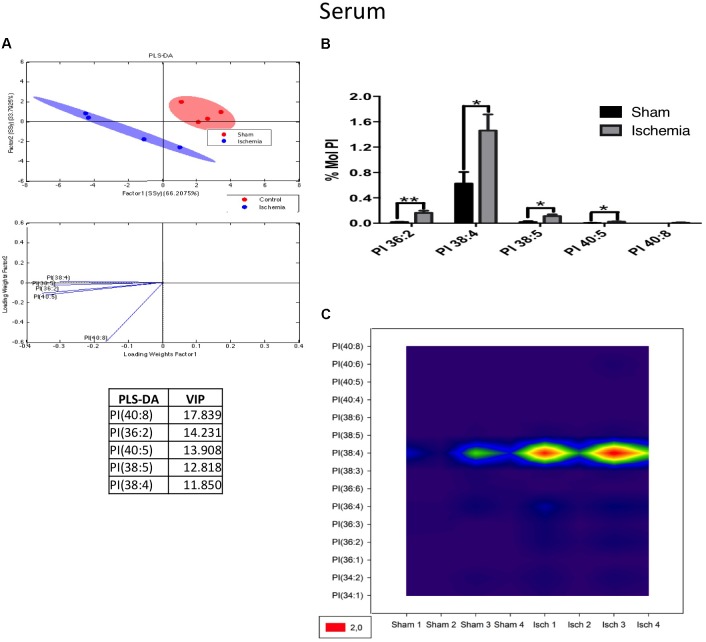
PI subspecies increased in the serum in postischemic rats. **(A)** Multivariate analyses: PLS-DA analysis used to discriminate between the lipid classes in the serum showing the factor score plots. **(B)** The PI profile is expressed as % mol composition. **(C)** Contour plots of the more influential subclasses of PI (variables) in the discriminant analysis for each evaluated variable. Data from the ischemic group were significantly different from those from the control group and are represented as the means ± SEM (^∗^*p* < 0.05 Student’s *t*-test for parametric data or the Mann–Whitney test for nonparametric data, *n* = 4 per group).

For its part, the following PI subspecies demonstrated increased levels in the ischemic group: PI 36: 2 (18: 1/18: 1); PI 38: 4 (18: 0/20: 4), and PI 38: 5 (18: 1 and 20: 4), with PI 38:4 being the most abundant according to the histogram and counter plot analyses (Figures 10A–C). Interestingly, PLs composed of fatty acids with long carbon chains, such as 18: 0 (stearic acid) 18: 1 (oleic acid) and 20: 4 (AA), are involved in proinflammatory processes and were detected in the serum of ischemic rats a month postinjury; maybe these results could be in relationship with the general increase of detected C 18:2 in TG and FFA fractions (Supplementary Figures S1E,F), future analysis should be done.

## Discussion

Novelty, this study described hippocampal and peripheral phospholipid profile changes in long-term postischemia associated with cognitive impairment in rats. The main changes on PLs were associated to hippocampal dysfunction, represented by a downregulation of PC, as precursor of acetylcholine and inverse levels of LPE and PS associated with peroxisome damage, a proinflammatory environment and cell death in the hippocampal parenchyma after 1 month of anoxia, glutamate excitotoxicity and Brain Blood Barrier (BBB) disruption generated by global ischemia in rats (Becerra-calixto and Cardona-gómez, 2017). Also, these findings are supported by our recent study were proinflammatory phospholipid profile was associated to neurodegeneration and neurological dysfunction (Marosi et al., 2006).

Our data suggested that spatial learning and reference memory were significantly impaired in global ischemic rats, a finding that was in line with those of previous studies (Deng et al., 2017). These results demonstrated that the damage in ischemic rats is large, and the functional outcome can become worse over time. Recently, the high incidence of cognitive impairment after an ischemic stroke event has been described (Sun et al., 2014) and the comorbidity factors, such as atherosclerosis (Knopman et al., 2016). Additionally, plasma phospholipid changes have been suggested in cognitive impairment associated with brain stroke patients but have not been clearly identified by the variability in humans (Li et al., 2016). However, in subcortical ischemic vascular dementia and mixed dementia, an adaptative increase in polyunsaturated fatty acids and elevated membrane degradation (Lam et al., 2014) have been observed. With respect to mild cognitive impairment and its progression to dementia, this condition has been addressed in Alzheimer-type dementia, with lipidomic brain changes in serum PLs being observed, mainly via reductions in PC (Wood et al., 2016). The findings of these studies are in accordance with our current data from an experimental model of global ischemia, however, we showed a specific fatty acid composition imbalance constituted of 18:0, 18:1, 20:4, and 22:6 in the parenchyma and peripheral PLs profile changes in long-term postischemia associated with cognitive impairment. The fatty acid composition of imbalanced PLs in familiar and sporadic Alzheimer’s disease in human brains has been commonly found, and in old triple transgenic AD mice with cognitive impairment, the imbalance has mainly been observed in PC and/or LPC, PE, and LPE (Villamil-Ortiz et al., 2016; Villamil-Ortiz et al., 2017).

Until now, few studies have focused on the impact of global ischemia on lipid signaling. Though some of these studies have investigated the first hours to 1 week of the acute phase of postischemia (Adibhatla and Hatcher, 2007), few have evaluated lipid profiles in the brain or serum, and none have focused on the potential relationship of these profiles with cognitive impairment. Therefore, our data are valuable for the detection of 12 lipid species, six of which had significant concentration changes in the hippocampus and serum, specifically PC, LPE, and PS in the hippocampus and PC, LPC, ePC and PI in the serum of ischemic and cognitively impaired rats during long-term postischemia, possibly suggesting differential lipid signatures under pathological conditions due to ischemia, which has been suggested by some related neurological studies (Lin and Perez-Pinzon, 2013; Shen et al., 2014; Hamazaki et al., 2016).

Glycerophospholipids are multifunctional molecules, are the major constituents of membranes and are responsible for the membrane bilayer, “mainly via choline or ethanolamine and to a lesser extent, inositol, serine or rarely, threonine. Further diversity is introduced by the components at the sn-1 and sn-2 positions, composing subclasses of diacyl and ether GP. Although plasmalogens represent up to 20% of the total phospholipid mass in humans, their physiological roles have been challenging to identify and are likely to vary in different tissues, metabolic processes and developmental stages” (Braverman and Moser, 2012). As plasmalogens serve as storage depots for second messengers and their precursors, membrane activity and ion channels, the study of plasmalogens may also provide insight into neural membrane pathology (Farooqui and Horrocks, 2007).

In particular, PC are composed of fatty acids with polyunsaturated chains, such as 34:2, 34:3, 36:4, 36:5, and 38:3, which are composed of linoleic acid (18:2), a precursor of the biosynthesis of AA (20:4) or AA composition *per se* (Choque et al., 2014), and may be supported by the increased LPC (18:0) catabolism to PC after ischemia. Additionally, elevated LPCs have been related to pathological lipid breakdown and the state of parenchymal inflammation after ischemia as an important source of reactive oxygen species (ROS) (Adibhatla and Hatcher, 2007; Wang et al., 2010), as well as being correlated with macrophage/microglia responses and neuronal death (Nielsen et al., 2016), spatial memory dysfunction (Köfeler et al., 2010) and its efflux and transport by ABCA7 in dementia by AD (Tomioka et al., 2017), also serving as a strategy in the forecast of ischemic stroke (Jickling and Montaner, 2015).

Furthermore, the lipid alterations found in our study suggested that phospholipid- modulating enzymes could be dysregulated in the brain, either due to increased or decreased phospholipase activity catalyzing the hydrolysis of LPC into PC. “The enzymes that catalyze the breakdown of PC to phosphatide (the phospholipase D or PLD enzymes) or to glycerophosphocholine and FFA (phospholipase A2 or PLA2 enzymes) have been directly associated with cerebral ischemia. In addition, alterations in the reaction cascades of PLD enzymes, leading to aberrant phosphatidic acid (PA) signaling, have been linked to neurodegenerative processes, with the activation of PLA2-family enzymes by β-amyloid peptide in neurons, in turn releasing secondary lipid messengers, such as AA. PLA2s also play a role in the modification of physical properties, such as the fluidity of the cellular membrane” (Whiley et al., 2014). It is accepted that, during ischemia and reperfusion, free fatty acid concentrations increase, particularly those of polyunsaturated fatty acids released from membrane PLs through activation of PLA2 (Hamazaki and Kim, 2013).

Another phospholipid that is highly involved is PS is the major acidic phospholipid in human membranes and one that constitutes 2–20% of the total phospholipid mass of adult human plasma and intracellular membranes (Van Meer et al., 2008). Hence, the presence of PS is essential for maintaining cell homeostasis, however, the fatty acid composition of PS is important, with studies of healthy human brains reporting that approximately 20–30% of the PS in human gray matter is composed of DHA (22:6), which is widely reported to be a pro-cell-survival fatty acid in the brain (Tanaka et al., 2012). Therefore, a reduction in the DHA content of PS is associated with the progression of mild cognitive impairment to dementia (Cunnane et al., 2012), possibly because the DHA in the PS conformation is essential for neuroprotection (Zhang et al., 2015). Additionally, PS synthesis may be inhibited by metabotropic glutamate receptor agonists, indicating that metabotropic glutamate receptor stimulation decreases not only the incorporation of serine in PS but also modulates the generation of excitatory postsynaptic currents in rat cerebellar slices. In addition, in neural membrane, PS modulates long-lasting changes in learning and memory according to the membrane composition (Farooqui and Horrocks, 2007). Interestingly, in our study, the increased PS 1 month postischemia was mainly composed of polyunsaturated fatty acids, such as AA 20: 4 (36:4, 38:4, 40:4), possibly suggesting an imbalance between the DHA and AA concentrations in PS. In the context of cerebral ischemia, excess intracellular calcium (Ca_i_^2+^) activates various lipases, including (PLA2) and PLC, which breakdown both intracellular and membrane phospholipids and release AA, thereby enhancing the proinflammatory response (Wang et al., 2007).

Interestingly, we also observed an increase in different species of LPE in the hippocampus of ischemic rats. LPE can be generated from PE via a phospholipase A-type reaction (Farooqui et al., 2000). Currently, the physiological significance of LPE in the brain after global ischemia is unknown. However, increased LPE has been demonstrated in major depressive disorder (MDD) and chronic stress (Liu et al., 2016; Oliveira et al., 2016). It has been reported that LPE has a direct relationship with calcium influx, which is closely related to cell death in neurodegenerative diseases and contributes to the cognitive impairment in transgenic mice with AD (Villamil-Ortiz et al., 2017).

For its part, phosphatidylinositol (PI) is characterized by the phosphorylation of the inositol head group of phosphoinositide, with a rapid and reversible phosphorylation rate, which critically participates in signal cascades and intracellular membrane trafficking (Hammond and Balla, 2015). PI is produced in the ER where its synthesizing enzymes, namely PI synthase (PIS) and CDP-DG synthase (CDS), are located (Kim et al., 2011). Based on the fact that PI is converted to PI3P in early endosomes and PI4P in the Golgi, plasma membrane (PM), and early and late endosomes, it is assumed that PI must be present in all of these membranes, as it has an important role in signaling pathways (Hammond and Balla, 2015). However, one past article mentioned that the four-vessel occlusion model-stimulated [3H]Inositol monophosphate formation via excitatory amino acids was greatly enhanced in hippocampal slices 24 h or 7 days after reperfusion (Seren et al., 1989). Additionally, PI has been recently reported to be a predictive marker of ischemic stroke (Tu et al., 2014).

In summary, our data showed that cognitive impairment in long-term postischemia is associated with a hippocampal phospholipid signature that indicates imbalance, proinflammatory environment, excitotoxicity and cell death, as evidenced in the peripheral PL profile related to cerebrovascular disruption and proinflammatory signaling and concomitantly supporting biomarkers of neurogenerative and cognitive impairment state and progression to long-term postischemia. Finally, this study provided a frame of reference for phospholipids that could be targeted for therapeutic exploration either through pharmacological intervention or enzymatic control, as phospholipases, for example, require further evaluation. Additionally, our findings are potentially useful for improving prediction and intervention after cerebral ischemia and form the basis for a future understanding of phospholipid dysfunction in neurological pathologies and targeting for prevention.

## Author Contributions

AS-G designed and realized the experiments, analyzed the data, wrote the paper. JV-O analyzed the data and wrote the paper. JA-L analyzed the data and reviewed the manuscript preparation. GC-G designed, analyzed and interpreted the data, prepared the manuscript, and critical revision. All authors read and approved the final manuscript.

## Conflict of Interest Statement

The authors declare that the research was conducted in the absence of any commercial or financial relationships that could be construed as a potential conflict of interest.

## Supplementary Material

FIGURE S1 Total changes in lipid fractions from hippocampus and serum after global ischemia. Cholesterol esters **(A,D)** triglycerides **(B,E),** free fatty, acid **(C,F)** from hippocampus and serum, respectively, from ischemic and control rats are shown. Individual concentrations of lipids are expressed as molar percentage. Myristic acid (14:0), palmitic acid (16:0), margaric acid (17:0), stearic acid (18:0), oleic acid (18:1), linoleic acid (18:2), arachidic acid (20:0), arachidonic acid (20:4), Erucic acid (22:1), Lignoceric acid (24:0), nervonic acid (24:1), docosahexaenoic, acid (22:6). Data represent means SEM of 4 mice per group. ^∗^*p* < 0.05, ^∗∗^*p* < 0.01, and ^∗∗∗^*p* < 0.001; for differences between sham and control groups.

## Abbreviations

ePC: ether phosphatidylcholine
ePE: ether phosphatidylethanolamine
ePS: ether phosphatidylserine
LPC: lysophosphatidylcholine
LPE: lysophosphatidylethanolamine
PA: phosphatidic acid
PC: phosphatidylcholine
PCA: principal component analyses
PE: phosphatidylethanolamine
PG: phosphatidylglycerol
PI: phosphatidylinositol
PLS-DA: partial least squares analysis
PS: phosphatidylserine
SM: sphingomyelin

## References

[B1] Adibhatla R. M. Hatcher J. F. (2007). Role of lipids in brain injury and diseases. *Future Lipidol.* 2 403–422. 10.2217/17460875.2.4.403 18176634 PMC2174836

[B2] Barker M. Rayens W. (2003). Partial least squares for discrimination. *J. Chemom.* 17 166–173. 10.1002/cem.785

[B3] Becerra-calixto A. Cardona-gómez G. P. (2017). Neuroprotection induced by transplanted CDK5 knockdown astrocytes in global cerebral ischemic rats. *Mol*. *Neurobiol.* 54 6681–6696. 10.1007/s12035-016-0162-2 27744570

[B4] Bermúdez-cardona J. Velásquez-rodríguez C. (2016). Phospholipids, cholesterol esters and triglycerides metabolic syndrome. *Nutrients* 8 1–13. 10.3390/nu8020054 26891317 PMC4772025

[B5] Billah M. M. Anthes J. C. (1990). The regulation and cellular functions of phosphatidylcholine hydrolysis. *Biochem. J.* 269 281–291. 10.1042/bj2690281 2201284 PMC1131573

[B6] Braverman N. E. Moser A. B. (2012). Functions of plasmalogen lipids in health and disease. *Biochim. Biophys. Acta – Mol. Basis Dis.* 1822 1442–1452. 10.1016/j.bbadis.2012.05.008 22627108

[B7] Cardona-Gómez G. P. Lopera F. (2016). Dementia, preclinical studies in neurodegeneration and its potential for translational medicine in South America. *Front. Aging Neurosci.* 8:304. 10.3389/fnagi.2016.00304 28066230 PMC5167748

[B8] Choque B. Catheline D. Rioux V. Legrand P. (2014). Linoleic acid: between doubts and certainties. *Biochimie* 96 14–21. 10.1016/j.biochi.2013.07.012 23900039

[B9] Cunnane S. C. Schneider J. A. Tangney C. Tremblay-Mercier J. Fortier M. Bennett D. A. (2012). Plasma and brain fatty acid profiles in mild cognitive impairment and Alzheimer’s disease. *J. Alzheimers Dis.* 29 691–697. 10.3233/JAD-2012-110629.Plasma 22466064 PMC3409580

[B10] Deng M. Xiao H. Zhang H. Peng H. Yuan H. Xu Y. (2017). Mesenchymal stem cell-derived extracellular vesicles ameliorates hippocampal synaptic impairment after transient global ischemia. *Front. Cell. Neurosci.* 11:205. 10.3389/fncel.2017.00205 28769765 PMC5511812

[B11] Farooqui A. A. Horrocks L. A. (eds) (2007). “Phospholipases A2 in neurological disorders,” in *Glycerophospholipids in Brain* (New York, NY: Springer), 394. 10.1007/978-0-387-49931-4

[B12] Farooqui A. A. Horrocks L. A. Farooqui T. (2000). Glycerophospholipids in brain: their metabolism, incorporation into membranes, functions, and involvement in neurological disorders. *Chem. Phys. Lipids* 106 1–29. 10.1016/S0009-3084(00)00128-6 10878232

[B13] Hamazaki K. Kim H. Y. (2013). Differential modification of the phospholipid profile by transient ischemia in rat hippocampal CA1 and CA3 regions. *Prostaglandins Leukot. Essent. Fat. Acids* 88 299–306. 10.1016/j.plefa.2013.01.003 23395327 PMC3622766

[B14] Hamazaki K. Maekawa M. Toyota T. Iwayama Y. Dean B. Hamazaki T. (2016). Fatty acid composition and fatty acid binding protein expression in the postmortem frontal cortex of patients with schizophrenia: a case-control study. *Schizophr. Res.* 171 225–232. 10.1016/j.schres.2016.01.014 26792082

[B15] Hammond G. R. Balla T. (2015). Biochimica et biophysica acta polyphosphoinositide binding domains?: key to inositol lipid biology ?. *Biochim. Biophys. Acta* 1851 746–758. 10.1016/j.bbalip.2015.02.013 25732852 PMC4380703

[B16] Jickling G. C. Montaner J. (2015). Lysophosphatidylcholine to stratify risk of ischemic stroke in TIA. *Neurology* 84 17–18. 10.1212/WNL.0000000000001100 25471396

[B17] Jordi Folch S. S. (1957). A simple method for isolation and purification of total lipides from animal tissues. *J. Biol. Chem.* 226 497–509. 10.1016/j.ultrasmedbio.2011.03.005 13428781

[B18] Kim Y. J. Guzman M. L. Balla T. (2011). A highly dynamic ER-derived phosphatidylinositol synthesizing organelle suplies phosphoinositides to cellular membranes. *Dev. Cell* 21 813–824. 10.1016/j.devcel.2011.09.005.A 22075145 PMC3235737

[B19] Knopman D. S. Gottesman R. F. Sharrett A. R. Wruck L. M. Windham B. G. Coker L. (2016). Mild cognitive impairment and dementia prevalence: the atherosclerosis risk in communities neurocognitive study, *Alzheimer’s Dement.* 2 1–11. 10.1016/j.dadm.2015.12.002 26949733 PMC4772876

[B20] Köfeler H. C. Tiboldi A. Hoeger H. Lubec G. (2010). Hippocampal lipids linked to spatial memory in the C57bl/6j mouse. *Neurochem. Int.* 57 935–939. 10.1016/j.neuint.2010.09.013 20933031

[B21] Lam S. M. Wang Y. Duan X. Wenk M. R. Kalaria R. N. Chen C. P. (2014). The brain lipidomes of subcortical ischemic vascular dementia and mixed dementia. *Neurobiol. Aging.* 35 2369–2381. 10.1016/j.neurobiolaging.2014.02.025 24684787 PMC4099521

[B22] Li D. Misialek J. R. Boerwinkle E. Gottesman R. F. Sharrett A. R. Mosley T. H. (2016). Plasma phospholipids and prevalence of mild cognitive impairment and/or dementia in the ARIC neurocognitive study (ARIC-NCS). *Alzheimer’s Dement.* 3 73–82. 10.1016/j.dadm.2016.02.008 27408938 PMC4925799

[B23] Lin H. W. Perez-Pinzon M. (2013). The role of fatty acids in the regulation of cerebral vascular function and neuroprotection in Ischemia. *CNS Neurol. Disord.* 12 316–324. 10.2174/1871527311312030005 23469852

[B24] Liu X. Li J. Zheng P. Zhao X. Zhou C. Hu C. (2016). Plasma lipidomics reveals potential lipid markers of major depressive disorder. *Anal. Bioanal. Chem.* 408 6497–6507. 10.1007/s00216-016-9768-5 27457104

[B25] Llinas R. Barbut D. Caplan L. R. (2000). Neurologic complications of cardiac surgery. *Prog. Cardiovasc. Dis.* 43 101–112. 10.1053/pcad.2000.9030 11014328

[B26] Marosi M. Rákos G. Robotka H. Németh H. Sas K. Kis Z. (2006). Hippocampal (CA1) activities in Wistar rats from different vendors. Fundamental differences in acute ischemia. *J. Neurosci. Methods* 156 231–235. 10.1016/j.jneumeth.2006.03.010 16621009

[B27] Martinez-Gardeazabal J. González de San Román E. Moreno-Rodríguez M. LLorente-Ovejero A. Manuel I. Rodríguez-Puertas R. (2017). Lipid mapping of the rat brain for models of disease. *Biochim. Biophys. Acta* 1859 1548–1557. 10.1016/j.bbamem.2017.02.011 28235468

[B28] Miyawaki S. Imai H. Hayasaka T. Masaki N. Ono H. Ochi T. (2016). Imaging mass spectrometry detects dynamic changes of phosphatidylcholine in rat hippocampal CA1 after transient global ischemia. *Neuroscience* 322 66–77. 10.1016/j.neuroscience.2016.02.013 26873001

[B29] Moskowitz M. A. Lo E. H. Iadecola C. (2010). The science of stroke: mechanisms in search of treatments. *Neuron* 67 181–198. 10.1016/j.neuron.2010.07.002 20670828 PMC2957363

[B30] Nielsen M. M. B. Lambertsen K. L. Clausen B. H. Meyer M. Bhandari D. R. Larsen S. T. (2016). Mass spectrometry imaging of biomarker lipids for phagocytosis and signalling during focal cerebral ischaemia. *Sci. Rep.* 6:39571. 10.1038/srep39571 28004822 PMC5177920

[B31] Nucera A. Hachinski V. (2018). Cerebrovascular and Alzheimer disease: fellow travelers or partners in crime? *J. Neurochem.* 144 513–516. 10.1111/jnc.14283 29266273

[B32] Oliveira T. G. Chan R. B. Bravo F. V. Miranda A. Silva R. Zhou B. (2016). The impact of chronic stress on the rat brain lipidome, *Mol. Psychiatry* 21 80–88. 10.1038/mp.2015.14 25754084 PMC4565780

[B33] Phillis J. W. O’Regan M. H. (2004). A potentially critical role of phospholipases in central nervous system ischemic, traumatic, and neurodegenerative disorders. *Brain Res. Rev.* 44 13–47. 10.1016/j.brainresrev.2003.10.002 14739001

[B34] Schaller B. Graf R. (2004). Cerebral ischemia and reperfusion: the pathophysiologic concept as a basis for clinical therapy. *J. Cereb. Blood Flow Metab.* 24 351–371. 10.1097/00004647-200404000-00001 15087705

[B35] Seren M. S. Aldinio C. Zanoni R. Leon A. F. (1989). Stimulation of inositol phospholipid hydrolysis by excitatory amino acids is enhanced in brain slices from vulnerable regions after transient global Ischemia. *J. Neurochem.* 53 1700–1705. 10.1111/j.1471-4159.1989.tb09233.x 2572678

[B36] Shen H. Zhou J. Shen G. Yang H. Lu Z. Wang H. (2014). Correlation between serum levels of small, dense low-density lipoprotein cholesterol and carotid stenosis in cerebral infarction patients (65 years of age). *Ann. Vasc. Surg.* 28 375–380. 10.1016/j.avsg.2013.01.029 24200130

[B37] Sun J. H. Tan L. Yu J. T. (2014). Post-stroke cognitive impairment: epidemiology, mechanisms and management. *Ann. Transl. Med.* 2:80. 10.3978/j.issn.2305-5839.2014.08.05 25333055 PMC4200648

[B38] Tanaka K. Farooqui A. A. Siddiqi N. J. Alhomida A. S. Ong W. Y. (2012). Effects of docosahexaenoic acid on neurotransmission. *Biomol. Ther.* 20 152–157. 10.4062/biomolther.2012.20.2.152 PMC3792211 24116288

[B39] Tian H. Qiu T. Zhao J. Li L. Guo J. (2009). Sphingomyelinase-induced ceramide production stimulate calcium-independent JNK and PP2A activation following cerebral ischemia. *Brain Inj.* 23 1073–1080. 10.3109/02699050903379388 19891536

[B40] Tomioka M. Toda Y. Mañucat N. B. Akatsu H. Fukumoto M. Kono N. (2017). Lysophosphatidylcholine export by human ABCA7. *Biochim. Biophys. Acta* 1862 658–665. 10.1016/j.bbalip.2017.03.012 28373057

[B41] Tu W. J. Liu X. Y. Dong H. Yu Y. Wang Y. Chen H. (2014). Phosphatidylinositol-3,4,5-trisphosphate 5-phosphatase 1: a meaningful and independent marker to predict stroke in the Chinese population. *J. Mol. Neurosci.* 52 507–514. 10.1007/s12031-013-0206-2 24352714

[B42] Van Meer G. Voelker D. R. Feigenson G. W. (2008). Membrane lipids: where they are and how they behave. *Nat. Rev. Mol. Cell Biol.* 9 112–124. 10.1038/nrm2330 18216768 PMC2642958

[B43] Vijayan M. Kumar S. Bhatti J. S. Reddy P. H. (2017). *Molecular Links and Biomarkers of Stroke, Vascular Dementia, and Alzheimer’s Disease*. 1st Edn. Amsterdam: Elsevier Inc.,. 10.1016/bs.pmbts.2016.12.014 28253992

[B44] Villamil-Ortiz J. G. Barrera-Ocampo A. Arias-Londoño J. D. Villegas A. Lopera F. Cardona-Gómez G. P. (2017). Differential pattern of phospholipid profile in the temporal cortex from E280A-familiar and sporadic Alzheimer’s disease brains. *J. Alzheimer’s Dis.* 61 209–219. 10.3233/JAD-170554 29125487

[B45] Villamil-Ortiz J. G. Barrera-Ocampo A. Piedrahita D. Velásquez-Rodríguez C. M. Arias-Londoño J. D. Cardona-Gómez G. P. (2016). BACE1 RNAi restores the composition of phosphatidylethanolamine-derivates related to memory improvement in aged 3xTg-AD mice. *Front. Cell. Neurosci.* 10:260. 10.3389/fncel.2016.00260 27891075 PMC5105502

[B46] Wang H. Y. Liu C. B. Wu H. W. Kuo J. S. (2010). Direct profiling of phospholipids and lysophospholipids in rat brain sections after ischemic stroke. *Rapid Commun. Mass Spectrom.* 24 2057–2064. 10.1002/rcm 20552694

[B47] Wang Q. Tang X. N. Yenari M. A. (2007). The inflammatory response in stroke. *J. Neuroimmunol.* 184 53–68. 10.1016/j.jneuroim.2006.11.014 17188755 PMC1868538

[B48] Whiley L. Sen A. Heaton J. Proitsi P. García-Gómez D. Leung R. (2014). Evidence of altered phosphatidylcholine metabolism in Alzheimer’s disease. *Neurobiol. Aging.* 35 271–278. 10.1016/j.neurobiolaging.2013.08.001 24041970 PMC5866043

[B49] Wood P. L. Locke V. A. Herling P. Passaro A. Vigna G. B. Volpato S. (2016). Targeted lipidomics distinguishes patient subgroups in mild cognitive impairment (MCI) and late onset Alzheimer’s disease (LOAD). *BBA Clin.* 5 25–28. 10.1016/j.bbacli.2015.11.004 27051586 PMC4802395

[B50] Zhang W. Liu J. Hu X. Li P. Leak R. K. Gao Y. (2015). n-3 polyunsaturated fatty acids reduce neonatal hypoxic/ischemic brain injury by promoting phosphatidylserine formation and Akt signaling. *Stroke* 46 2943–2950. 10.1161/STROKEAHA.115 26374481

