# Supplementary figures and images for: Inverse Phosphatidylcholine/Phosphatidylinositol Levels as Peripheral Biomarkers and Phosphatidylcholine/Lysophosphatidylethanolamine-Phosphatidylserine as Hippocampal Indicator of Postischemic Cognitive Impairment in Rats
Source: Front Neurosci. 2018 Dec 21;12:989. doi: 10.3389/fnins.2018.00989 (PMC6309919; doi:10.3389/fnins.2018.00989)

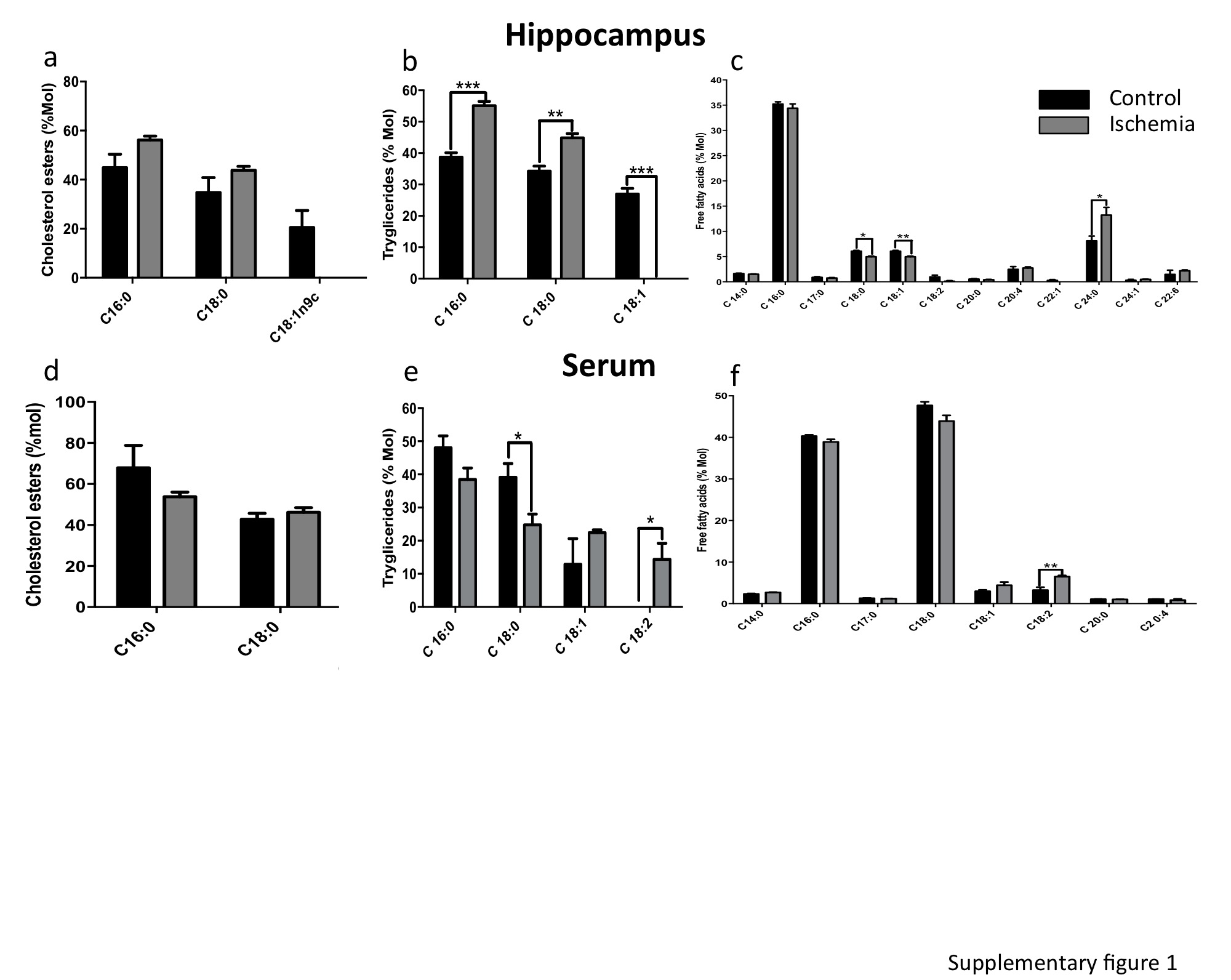

Supplement: FIGURE S1 — Total changes in lipid fractions from hippocampus and serum after global ischemia. Cholesterol esters (A,D) triglycerides (B,E), free fatty, acid (C,F) from hippocampus and serum, respectively, from ischemic and control rats are shown. Individual concentrations of lipids are expressed as molar percentage. Myristic acid (14:0), palmitic acid (16:0), margaric acid (17:0), stearic acid (18:0), oleic acid (18:1), linoleic acid (18:2), arachidic acid (20:0), arachidonic acid (20:4), Erucic acid (22:1), Lignoceric acid (24:0), nervonic acid (24:1), docosahexaenoic, acid (22:6). Data represent means SEM of 4 mice per group. ∗p < 0.05, ∗∗p < 0.01, and ∗∗∗p < 0.001; for differences between sham and control groups. [file Image_1.JPEG]
